# On Modern Progress in Ophthalmic Medicine and Surgery

**Published:** 1895-05-25

**Authors:** Robert Brudenell Carter

**Affiliations:** Consulting Ophthalmic Surgeon to St. George's Hospital


### On Modern Progress in Ophthalmic Medicine and Surgery.

By Robert Brudenell Carter, F.R.C.S., Consulting Ophthalmic Surgeon to St. George's Hospital.

### DISEASES OF THE CORNEA.

(*Continued from page* 75.)

The cornea is subject to various forms of inflammation, some of which are so rare as to be among the curiosities of ophthalmic practice, while others are sufficiently common, and sufficiently serious in their consequences, to deserve the best attention of every practitioner. Among the latter, two forms stand out pre-eminently, the so-called "vascular keratitis," and the "interstitial" keratitis, which is so closely associated with Mr. Hutchinson's discovery of its true nature. Both forms are seen more frequently in hospital practice than among persons who are comparatively well-to-do, and both occur far more frequently in childhood or youth than at any later period of life.

"Vascular" keratitis commences with some symptoms, at first seldom severe, of irritation of one of the eyes, with a little flushing of the conjunctiva, lacrymation, and discomfort. At this stage, the upper margin of the cornea may be seen to be invaded by an abnormal development of closely packed minute blood vessels, forming a vascular patch usually of crescentic outline, its widest part on the vertical meridian of the cornea, and its extremities tapering off to points. The vessels are so small, and so crowded together, that a strong magnifier is required in order to resolve the red patch into its constituent elements; and, when examined in profile, it will be seen to be distinctly elevated above the level of the corneal tissue, to which it descends by a perceptible marginal slope. This slope, looked at in front, is a greyish line, formed by epithelial disturbance. The crescent gradually increases both in length and width, and extends downwards over the cornea, preceded by its precursory grey line; while, before it has made much progress, a second crescent usually appears at the lower margin, precisely opposite to the first, and advances upwards to meet it. If the disease be suffered to proceed unchecked, the vascular crescents will, in time, meet and coalesce, until the whole cornea is of uniform redness, and vision for the time is entirely lost. At a period so recent, that it can hardly yet be called historical, this sequence of events was not uncommon; and there is a classical description of an "inflamed cornea" which compares it to "a ripe cherry, or a piece of red cloth." When this description is realised the cornea undergoes softening as well as vascularisation, with the result that its curvature is frequently modified by the pressure of the ocular muscles. Sooner or later—but generally not for many weeks, or even months—the red colour loses its vividness, the vessels dwindle and ultimately disappear, and leave behind them a dense white opacity, almost like white paper. Very slowly this opacity thins away and fades, first at the corneal margin, and then gradually towards its centre, so that, in favourable cases, something like useful vision may ultimately be restored. The disease, which usually commences in one eye, almost invariably attacks the other, although the second eye seldom suffers so severely as the first. Vascular keratitis, as I have seen it in hospital patients who had been neglected or maltreated at the outset, has often meant months of blindness, and a subsequent lifetime of seriously impaired vision.

The first principle of treatment is to do no mischief; and mischief, often irremediable, may be done by even a single application of any chemical irritant. The very bad cases are those in which the crescent has not been seen, perhaps not even looked for, in which the early stage has been regarded as a trivial conjunctivitis, and in which drops of nitrate of silver, or of sulphate of zinc, have been prescribed and used. The consequences of neglect may be bad enough; but neglect is far better than such treatment as this.

In hospital practice, many years ago, it was my lot to see several instances in which a cornea once completely opaque began to clear at the margin, and in some of these I performed iridectomy as soon as the clear margin was wide enough to allow the patient to see a little through the artificial pupil. It occurred to me that, as an iridectomy would ultimately be required in bad cases, it would be worth while to perform it in the acute stage, and the results of doing so were eminently satisfactory. While the proceeding was still tentative, I selected the worse eye of the two for operation, with the invariable result that it became the better of the two, had the lesser degree of opacity, and made the quicker and more complete recovery. Still, the cases left much to be desired, and I began to think the improvement was mainly due to the extent to which the iridectomy incision cut off the new vessels upon the cornea from their sources of supply. An obvious step farther was to defer the iridectomy, and to sever the vascular connection still more completely by peritomy; and I ultimately limited my dissection for this purpose to the portion of conjunctiva corresponding to each crescent. In time, the galvanic cautery seemed to offer a better means of effecting the severance than the scissors; and in fairly robust subjects I found the cure to be greatly promoted by the occasional application of a leech to the temple. It should be placed on the same horizontal line as the lid opening, and as far from the eye as the hair will allow.

The first thing, then, in a case of vascular keratitis, is to recognise the character of the affection, and the dangers which it involves. The patient or parents should be warned that the second eye will probably suffer, and should understand that the malady is serious enough to require the most careful attention. The state of the general health should be considered and treated, and all use of the eyes in reading or the like, and all exposure of them to heat, cold, or glare, should be strictly forbidden. Cocaine and atropine should be used, and the patient kept in a dim light. If, in twenty-four hours, the crescent is increasing, the eye should be thoroughly cocainised, and a heated Wordsworth's nævus needle, or a galvanic cautery, should be so used as to make an eschar completely cutting off the new vessels on the cornea from the tissues posterior to them. If there be any reaction, a leech may be applied as above directed, and repeated every other day if required. In the majority of cases, according to my experience, the further extension of the mischief will be at once arrested, and a short period of ordinarily prudent management will complete the cure.

